# Tonsillectomy

**Published:** 1909-05-08

**Authors:** Philip A. Harry


### The General Practitioner's Column.

[Contributions to this Column are invited, and if accepted will be paid for.]

### TONSILLECTOMY.

By PHILIP A. HARRY, M.D., D.P.H.

An article by Dr. Matthews, of New York, in a recent number of the *Annals of Surgery* * provides a suitable excuse for drawing further attention to the removal of tonsils by enucleation. The removal of a great number of tonsils by this method during the past year has shown its advantages over any other method. It is thoroughly efficient and so simple that it can be performed by the most newly appointed surgical dresser.

As far as enucleation is concerned, tonsils may be divided into two classes, the hard and the soft. Hard tonsils, large or small, of any size or shape, enucleate with the greatest ease. Soft tonsils, especially if they are also small, feeling like adenoids, are apt to be difficult. But their removal by guillotine is even more difficult. Fortunately they rarely call for enucleation: it is usually the opposite tonsil that is enlarged and has produced symptoms necessitating operation. There are many other methods of removing tonsils. Among others may be mentioned the several varieties of guillotine, which are certainly rapid, but not much more so than enucleation.

The guillotine leaves a cut surface from which hæmorrhage is very often profuse, and by which organisms find easy entrance to the body. Dr. Matthews has seen a first attack of follicular tonsillitis follow partial removal by the guillotine, and it is not at all uncommon to see septic throats after removal by this method. During and after enucleation there is usually a good deal of hæmorrhage, but this can be effectively controlled by a modification in the operation to be mentioned later.

Snares and cauteries which were introduced to prevent or lessen hæmorrhage are often dangerous and unsatisfactory. They require plenty of time and docile patients. Punches and scissors are incomplete. The pillars of the fauces and even the pharyngeal wall may be injured. The method of removing shavings by pulling the tonsil inwards with a special vulsellum and slicing off portions of varying sizes may appeal to some operators. Even with special bistouries damage may be done to the tongue and surrounding structures. The patient requires to be deeply anæsthetised, preferably with chloroform, whereas during enucleation anæsthesia may be very light. Pillar knives, separators, and hooks were introduced by some operators who removed the entire tonsil, not by touch but by sight. These instruments are quite superfluous. It is very rarely that the finger cannot break down the adhesions between the anterior and posterior pillars and the tonsils; indeed, it is rare to find adhesions.

The operation of enucleation can be done by the sense of touch alone. It is necessary to bear in mind a few anatomical facts. The tonsils are collections of lymphoid tissue forming part of the ring of Waldeyer, guarding the entrance to the pharynx and larynx. They are situated between the anterior and posterior pillars of the fauces, and have a more or less definite capsule. The majority of vessels enter and leave this very vascular organ at its lower part. The internal carotid is one inch external and posterior to the tonsil. Hæmorrhage comes from one or more enlarged tonsillar arteries: it must be remembered that all the important vessels in this region (lingual, facial, ascending pharyngeal, internal maxillary), send branches to the tonsil. For the operation about to be described the best anæsthetic is certainly ether or gas and ether. Chloroform is not safe enough, and ethyl chloride anæsthesia is too short. It may be mentioned here that a widely-opened gag may interfere with the breathing.

The patient should be on a fairly low table, the hair enclosed in a towel or rubber cap. The head remains on a level with the rest of the body. After the insertion of a gag, preferably in the left side of the mouth, the operator stands on the right side of the patient and inserts his left forefinger between the tonsil and the anterior pillar of the fauces. The finger is insinuated backwards and upwards until the external surface of the tonsil is reached, and until the upper half of the tonsil is separated. A small-sized Guy's tongue-forceps† is then taken in the right hand, from an assistant, and with the left forefinger as guide the partly-separated tonsil is seized. The separation is then continued downwards until the lower part of the organ is reached, the tongue forceps pulling gently inwards all the time and also being rotated to the right. It is this rotation to the right for two or three turns that is efficacious. By this manœuvre the largest vessels of the tonsil which enter towards its lower part are twisted and torn, thus considerably lessening the amount of hæmorrhage. This simple modification also greatly shortens the operation.

These remarks apply equally to either tonsil. The gag need not be changed from left side to right. It is not necessary, as Dr. Matthews recommends, to use the guillotine for the final separation: the operation is needlessly prolonged and there is a risk of the tonsil escaping down the pharynx, especially in the case of a deep or double tonsil. It is to prevent this accident that the tonsil is seized when only the upper half is free. The lowest part of the tonsil is always difficult to detach. In some cases there is quite a tough fibrous band to be torn through, and it is while endeavouring to do this that the organ slips away and hangs into the pharynx. Attempts to find it are not always successful; the act of deglutition usually completes the separation.

For small soft tonsils the assistant or nurse should exert pressure behind the angle of the jaw. It is best to remove this variety with the first and second fingers of the left hand working together, or, if

* December 1908.

† Down Bros.

possible, the first finger and thumb. If the tonsil is gripped by the tongue forceps the pull inwards should be very gentle; indeed, the forceps should merely be used to support the separated part.

After the operation is completed the nurse should sponge the patient's face with cold water. It cleans the face, stops hæmorrhage (especially if adenectomy has been done), and helps to bring the patient round from the anæsthetic.

Before he is quite round and while he is lying with his face over the side of the table the finger should be passed over the areas operated on; occasionally there is a small tonsil at the base of the tongue, below the one removed and entirely separate from it. It is advisable not to do more than commence its detachment with the finger. It is then easily removed with the Guy's tongue forceps.

If a tonsil is small and soft it may be torn; no object is gained in attempting to remove the small piece that remains. The advantages of tonsillectomy by enucleation are numerous. The operation is easily performed. The immediate and future results are equally gratifying. It is satisfactory to know that the entire organ has been removed and recurrence impossible. Second, and even third recurrences are not uncommon after the guillotine, bistoury, scissors, punch forceps, etc. Double tonsils are not missed, as they often are with the guillotine and bistoury. By the latter method of performing tonsillotomy not only may a small second tonsil be missed, but if the incision be made from below upwards the lower part of the tonsil may be left.

Enucleation effectively opens up the supratonsillar fossa—a favourite receptacle for septic matter. The raw surface left rapidly diminishes in size owing to the falling together of the pillars of the fauces. Decayed molar teeth are not so great a contra-indication to this operation as to tonsillotomy. Follicular tonsillitis, which may occur after removal of a portion of the tonsil, never occurs after enucleation. The wound heals very quickly. Enlarged glands diminish in size and gradually disappear after tonsillectomy.

The operation can be performed entirely by the sense of touch, and with only two instruments, including the gag. Hæmorrhage is reduced to a minimum by the manœuvre mentioned above. Tonsil hæmostats and styptics are uncalled for. Late hæmorrhage is unknown. Little or no after-treatment is required. Another important point is that the surgeon need not be ambidextrous. With the guillotine and bistoury the non-ambidextrous surgeon must stand behind the patient's head to remove the right tonsil.

The disadvantages of the method are mainly the patients' objections. A lady objected on religious grounds to having her child's tonsil removed by enucleation because she thought the organ was placed there by Providence for some definite purpose. She was, however, perfectly satisfied with the results obtained in an elder brother, who, unknown to her, had had his tonsils enucleated. An abnormal tonsil is always a source of danger, and if removal is called for it should be complete, especially as total removal is just as easy or even easier than partial. The objection that enucleation necessitates an anæsthetic need not be considered, seeing that partial removal by guillotine is always done under an anæsthesia of some kind even where there are no adenoids. Changes in the voice have been mentioned as a disadvantage of tonsillectomy. These changes can only be detected by the objectors themselves. One operator heard of someone who got a septic finger after performing tonsillectomy. The finger was scratched by the sharp teeth of the patient. This accident should not occur if care is taken to keep the gag in position.

